# Eosinophil–Epithelial Cell Crosstalk at Mucosal Barriers: From Homeostatic Regulation to Disease Pathogenesis

**DOI:** 10.3390/cells15090832

**Published:** 2026-05-01

**Authors:** Janet Lee, Eunsoo Kim

**Affiliations:** College of Pharmacy and Research Institute for Drug Development, Pusan National University, Busan 46241, Republic of Korea; janetlee@pusan.ac.kr

**Keywords:** eosinophils, epithelial cells, mucosal immunity, alarmins, type 2 immunity

## Abstract

Eosinophils are multifunctional granulocytes that reside constitutively within mucosal tissues, where they engage in bidirectional communication with the epithelial cells lining the respiratory and gastrointestinal (GI) tracts. Once regarded solely as terminal effectors of the type 2 immunity, eosinophils are now recognized as key regulators of epithelial homeostasis and barrier integrity. Epithelial cells initiate crosstalk by releasing the alarm cytokines such as interleukin (IL)-33, thymic stromal lymphopoietin (TSLP), and IL-25, which drive eosinophil recruitment, activation, and tissue retention. Conversely, eosinophils modulate epithelial function through the release of granule proteins, cytokines, and growth factors with both damaging and reparative consequences. In the airway, this crosstalk underpins the pathogenesis of eosinophilic asthma and chronic rhinosinusitis with nasal polyps (CRSwNP), in part via eosinophil-derived mediators that disrupt tight junction integrity and fuel remodeling. In the GI tract, homeostatic eosinophils support villous architecture, epithelial turnover, and goblet cell differentiation through microbiota-driven IL-33 signals and neuropeptide-mediated neuroimmune pathways, whereas dysregulated crosstalk promotes eosinophilic esophagitis (EoE) and inflammatory bowel disease (IBD). This review synthesizes recent research to delineate the molecular mechanisms of eosinophil–epithelial crosstalk across mucosal compartments, highlight tissue-specific differences and shared mechanistic themes, and discuss the implications of these findings for targeted therapy.

## 1. Introduction

Eosinophils are bone marrow-derived granulocytes that constitutively home to the gastrointestinal (GI) lamina propria and, to a lesser extent, the lung interstitium via the eotaxin-1/CCR3 chemokine axis [1,2]. Classically associated with anti-helminth immunity and allergic pathology, eosinophils have long been characterized as destructive end-effectors that accumulate at sites of the type 2 inflammation and release cytotoxic granule proteins upon activation [3,4]. However, an expanding body of research has fundamentally revised this view, demonstrating that eosinophils perform essential homeostatic functions at mucosal surfaces that are not limited to host defense [5,6].

Central to this reconceptualization is the recognition that eosinophils engage in bidirectional communication with the epithelial cells lining the respiratory and GI tracts. Epithelial cells serve as sentinels of environmental insults, releasing the alarm cytokines IL-33, TSLP, and IL-25, which recruit and activate eosinophils [7]. Conversely, activated eosinophils release a range of mediators that can both damage and repair the epithelial barrier, regulate epithelial differentiation, and orchestrate downstream immune responses [8,9]. This bidirectional axis operates across multiple disease states: in the airways, eosinophil–epithelial interactions drive features of eosinophilic asthma and chronic rhinosinusitis with nasal polyps (CRSwNP), including subepithelial fibrosis, mucus hypersecretion, and airway hyperresponsiveness (AHR) [10]; in the GI tract, the same axis governs villous integrity during homeostasis [11] while becoming dysregulated in eosinophilic esophagitis (EoE) and inflammatory bowel disease (IBD) [12,13].

Recent advances in single-cell transcriptomics, organoid co-culture systems, and conditional knockout models have begun to resolve the molecular language of eosinophil–epithelial crosstalk at cellular and molecular level [14,15]. Key studies have established that intestinal eosinophils are essential for microbiota-dependent villous homeostasis [11] and goblet cell differentiation [16], while in the esophagus, bidirectional co-culture experiments have uncovered the self-sustaining nature of EoE pathology [15]. Concurrently, the identification of novel eosinophil-derived mediators, including IL-24 [17] and eosinophil extracellular trap (EET)-associated galectin-10 [18], whose mechanisms and mucosal consequences are discussed in detail in Section 4.2 and Section 4.3, respectively. Together, these findings provide the mechanistic foundation for a unified framework of eosinophil–epithelial crosstalk across mucosal compartments.

## 2. Eosinophil Biology at Mucosal Sites

### 2.1. Development, Trafficking, and Tissue Residency

Eosinophil development is initiated in the bone marrow from granulocyte-macrophage progenitors (GMPs) and gives rise to the eosinophil lineage-committed progenitor (EoP), a stage identified by Iwasaki et al. as IL-5Rα+CD34+c-Kitlo cells capable of differentiating into eosinophils [19]. The transcription factors GATA-1 and GATA-2 are essential determinants of eosinophil lineage commitment: enforced GATA-1 expression in human myeloid progenitor cells switches cell fate toward the eosinophil lineage, and GATA-1-deficient fetal liver cells fail to produce eosinophil progenitors [20]. A high-affinity GATA-binding site in the GATA-1 promoter (dblGATA) is essential for eosinophil development in vivo, as its targeted deletion leads to selective ablation of the eosinophil lineage [21]. The resulting ΔdblGATA mouse model has been widely used to study eosinophil function in vivo.

Upon maturation, eosinophils exit the bone marrow and traffic to mucosal tissues via a CCR3–eotaxin axis [2]. Eotaxin-1 (CCL11), produced constitutively by intestinal epithelial cells and smooth muscle, provides the homeostatic gradient for baseline GI eosinophil homing [1,22]. During inflammation, eotaxin-2 (CCL24) and eotaxin-3 (CCL26), produced by activated epithelial cells, fibroblasts, and mast cells, amplify tissue eosinophilia through CCR3-dependent mechanisms [23]. IL-5 further enhances CCR3 responsiveness and prolongs eosinophil survival once they reach target tissues [24]. Ignacio et al. demonstrated that small intestinal (SI) eosinophils are recruited prenatally (before microbial colonization) and subsequently activated by microbiota-dependent epithelial IL-33 release to maintain villous architecture, epithelial barrier function, and intestinal macrophage maturation [11]. Eosinophil-deficient ΔdblGATA mice exhibited villus blunting, increased intestinal motility, and impaired lipid absorption, phenotypes rescued by adoptive transfer of bone-marrow-derived eosinophils [11].

### 2.2. Granule Architecture and Secretory Mechanisms

Mature eosinophils contain cytoplasmic specific granules with an electron-dense crystalline core composed primarily of major basic protein 1 (MBP-1) and a matrix rich in eosinophil cationic protein (ECP), eosinophil peroxidase (EPO), and eosinophil-derived neurotoxin (EDN) [3]. These cationic proteins act through distinct cytotoxic mechanisms. MBP-1 disrupts cell membrane integrity by interacting with anionic phospholipid bilayers. ECP forms pores in lipid bilayers, facilitating the entry of other toxic molecules. EPO generates reactive oxygen species via halide-dependent peroxidase reactions, damaging epithelial glycocalyx proteins [4,8].

Beyond granule proteins, eosinophils store and release preformed cytokines, including IL-4, IL-13, TGF-β1, TNF-α, and GM-CSF; lipid mediators (leukotriene C4, prostaglandin D2); and angiogenic factors (VEGF, FGF-2) [3]. The dominant secretory mechanism in eosinophils is piecemeal degranulation (PMD), a process distinct from classical exocytosis, in which small vesiculotubular carriers (termed eosinophil sombrero vesicles, EoSVs) bud from specific granules and transport granule contents to the cell surface for extracellular release [25]. Melo et al. demonstrated by electron tomography that eotaxin, RANTES, and platelet-activating factor stimulate the formation of intragranular vesiculotubular networks that mediate selective MBP-1 mobilization without complete granule exocytosis [25]. This selective release mechanism allows eosinophils to modulate local immune responses through differential cytokine secretion while preserving their capacity for rapid cytotoxic degranulation [26]. Pharmacological inhibition of these secretory pathways has provided important mechanistic insights into eosinophil degranulation. Brefeldin A, a Golgi-disrupting agent, collapses intragranular vesiculotubular networks and abolishes PMD-dependent release of MBP and cytokines, establishing vesicular transport as the essential conduit for selective granule protein secretion [27]. In contrast, monensin, a granule-targeting ionophore, permeabilizes the acidic secretory granules of eosinophils, elevating granule pH and triggering granzyme B-dependent, caspase-independent cell death selectively in eosinophils over neutrophils, suggesting its potential as a targeted eosinophil-depleting agent [28]. At the upstream level, suplatast tosilate (IPD-1151T), a selective Th2 cytokine inhibitor, suppresses T cell-derived IL-4 and IL-5 production, thereby reducing eosinophil tissue infiltration and granule protein release in allergic airway disease [29]. In addition to PMD, eosinophils can undergo cytolytic degranulation, in which cell death results in the extracellular release of intact, secretion-competent granules that retain the capacity to degranulate upon appropriate stimulation [30].

### 2.3. Eosinophil Heterogeneity: Tissue-Resident Subsets

A single-cell transcriptomic and proteomic atlas of mouse eosinophils across multiple tissues by Hu et al. revealed that eosinophil identity is shaped by tissue-derived cues and residency duration [31]. Long-lived eosinophils in the SI diversify into transcriptionally and phenotypically distinct subsets, whereas lung eosinophils remain comparatively uniform, suggesting that the GI microenvironment drives distinct functional specialization in resident eosinophils [31,32]. Kutyavin et al. subsequently demonstrated that nutrient-derived retinoic acid signals in the SI further shape eosinophil adaptation, regulating their surface marker expression and survival independently of cytokine stimulation [33].

In the SI, Li et al. identified a subset of eosinophils expressing neuromedin U receptor 1 (NMUR1) through transcriptome profiling and fate-mapping reporter mice [16]. The neuropeptide neuromedin U (NMU), produced by enteric neurons, activates these NMUR1-expressing eosinophils, which in turn promote goblet cell differentiation in intestinal organoid co-culture experiments and facilitate worm expulsion during helminth infection [16]. Notably, NMUR1 expression was programmed by the local SI microenvironment and further enhanced by inflammation, underscoring the role of tissue-specific cues in shaping eosinophil functionality [16]. The concept that eosinophils are wired for neuroimmune communication extends beyond the intestine. In the skin, a subset of prodynorphin-expressing sympathetic neurons recruits eosinophils through the CCL11-CCR3 axis and activates them via the beta2 adrenergic receptor, mediating stress-induced exacerbation of atopic dermatitis [34]. Although the skin lies outside the mucosal compartments addressed in this review, this finding reinforces the principle that local neuronal signals shape eosinophil activation state in a tissue-specific manner, a mechanism with direct parallels to the NMU-NMUR1 axis in the intestinal mucosa [16]. At a broader level, transcriptomic studies have proposed at least two functionally distinct subpopulations, inflammatory eosinophils (iEos) and regulatory eosinophils (rEos), whose relative abundance and activation state are shaped by both systemic and local tissue signals [35].

In the context of EoE, co-culture of human peripheral blood eosinophils with the esophageal epithelial cell line EPC2 demonstrated that epithelial-derived GM-CSF programs a distinct eosinophil phenotype with altered surface marker expression and cytokine profiles [15]. In a subsequent study, TGF-β exposure in esophageal epithelial co-culture conditions induced a CD62L-high regulatory eosinophil subset that promotes fibroblast activation and extracellular matrix (ECM) remodeling, and expresses transcripts associated with immune regulation and fibrosis, including TLR1, TLR6, VEGFB, and CD101 [36]. These data demonstrate that eosinophil heterogeneity is actively sculpted by epithelial signals, with significant consequences for disease outcomes. Underlying these tissue-specific adaptations, epigenetic mechanisms including DNA methylation, histone modifications, and noncoding RNAs have been shown to regulate eosinophil gene expression programs and contribute to their activation state in allergic and asthmatic inflammation, adding a further layer of regulation to the functional heterogeneity described above [37].

## 3. Epithelial Alarmin Signals: Initiating Eosinophil–Epithelial Crosstalk

### 3.1. Barrier Dysfunction as the Trigger

**Figure 1:**
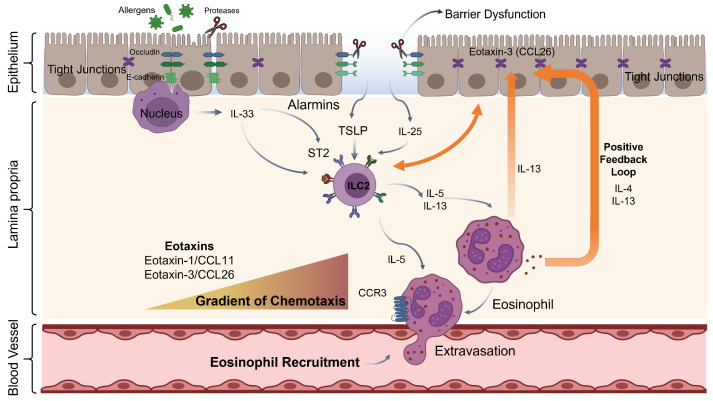
**Epithelial Alarmin Signaling and the ILC2–Eosinophil Amplification Circuit at Mucosal Barriers.** Barrier disruption triggers passive IL-33 release from epithelial nuclei and active secretion of TSLP and IL-25. These alarmins activate ILC2s via ST2, TSLPR, and IL-17RB, driving IL-5 and IL-13 production. IL-5 promotes eosinophil survival and CCR3 upregulation, while eotaxin-1 (CCL11) and eotaxin-3 (CCL26) establish a chemotactic gradient for eosinophil tissue recruitment. Activated eosinophils produce IL-4 and IL-13, sustaining a positive feedback loop. Disruption of tight junction proteins (occludin, E-cadherin) initiates the cascade. Abbreviations: TSLP, thymic stromal lymphopoietin; ILC2, group 2 innate lymphoid cell; ST2, IL-33 receptor; TSLPR, TSLP receptor; IL-17RB, IL-25 receptor; CCR3, C-C chemokine receptor type 3; CCL11, eotaxin-1; CCL26, eotaxin-3.

Disruption of the epithelial barrier is a key initiating event in eosinophil–epithelial crosstalk, exposing underlying immune cells to environmental triggers and activating epithelial alarm signaling (Figure 1). In the airway, allergens with protease activity cleave tight junction proteins, including occludin and E-cadherin, thereby directly disrupting paracellular permeability [7]. In the esophagus, loss-of-function mutations in the cornified envelope gene filaggrin (FLG) are associated with susceptibility to EoE, implicating defective barrier formation as a prerequisite for eosinophil accumulation [38]. Additionally, IL-13 produced downstream of eosinophil activation suppresses the expression of desmoglein-1 (DSG1), a desmosomal cadherin whose loss is a hallmark of esophageal barrier dysfunction in EoE, thereby creating a self-reinforcing loop of barrier failure and eosinophil recruitment [39]. In the SI, eosinophils actively contribute to maintaining epithelial barrier integrity, as their absence leads to measurable increases in epithelial permeability [11]. In IBD, conversely, persistent barrier dysfunction is associated with sustained eosinophil activation even during disease remission [13].

### 3.2. IL-33: The Nuclear Alarmin

Among the alarmins released upon epithelial barrier disruption, IL-33 is unique in its nuclear origin and passive release mechanism. IL-33 is constitutively expressed in the nuclei of epithelial and endothelial cells and is released passively upon cell necrosis, mechanical stress, or environmental protease-mediated activation [40]. Full-length IL-33 is further processed by neutrophil and mast cell proteases into truncated, potently active forms that signal through the receptor ST2 (IL1RL1), which is expressed on eosinophils, ILC2s, mast cells, and T cells [40]. In the gut, Ignacio et al. demonstrated that microbial colonization induces IL-33 release from intestinal epithelial cells, which subsequently activates resident eosinophils via ST2 to maintain villous architecture and macrophage maturation [11]. Eosinophil-deficient and ST2/IL17rb-deficient mice shared equivalent villous defects, establishing the microbiota–epithelial IL-33–eosinophil ST2 axis as the molecular backbone of homeostatic intestinal crosstalk [11]. IL-33 has also been reported to directly promote eosinophil survival, adhesion, and degranulation in a ST2-dependent manner, establishing eosinophils as direct IL-33 targets independent of ILC2 intermediaries [41].

In the lung, Alhallak et al. demonstrated that mast-cell-derived prostaglandin E2 (PGE2) drives production of the soluble IL-33 decoy receptor sST2 from mast cells, thereby limiting IL-33 bioavailability and suppressing tissue eosinophilia [42]. Genetic loss of this brake in mast cell-specific IL1RL1-deficient mice resulted in exaggerated lung eosinophilia, ILC2 expansion, and amplified airway inflammation, indicating that mast cell-eosinophil crosstalk through the PGE2-sST2-IL-33 axis critically sets the threshold for eosinophilic airway disease [42].

### 3.3. TSLP and IL-25

While IL-33 signals across both the GI tract and airway, two additional epithelial cytokines, TSLP and IL-25, complement and amplify alarmin-mediated eosinophil recruitment across mucosal compartments. TSLP is an epithelial cytokine produced in response to diverse stimuli, including allergens, detergents, microbial products, and mechanical injury [7]. It drives type 2 immune priming by activating dendritic cells and basophils [43], and by stimulating ILC2s to release IL-5, thereby amplifying eosinophil survival and activation [7]. In EoE, Noti et al. demonstrated that TSLP expressed by esophageal epithelial cells promotes basophil-driven esophageal eosinophilia [44]. TSLP-elicited basophil responses were required for eosinophil accumulation in a murine food allergen-induced EoE model, revealing an unexpected basophil intermediary in the TSLP–eosinophil axis [44]. In the airway, TSLP similarly drives eosinophilic inflammation in asthma and CRSwNP through ILC2-dependent amplification of IL-5 signaling [7], a mechanism explored further in Section 5 of this review. Complementing TSLP, IL-25 (IL-17E), expressed by and released from epithelial cells upon protease exposure, reinforces the ILC2–eosinophil circuit, particularly during helminth expulsion and chronic allergic disease [7]. Buonomo et al. demonstrated that IL-25 is a microbiota-regulated signal that protects against lethal Clostridioides difficile infection in an eosinophil-dependent manner, underscoring the role of epithelial IL-25–eosinophil communication in intestinal pathogen defense [45].

### 3.4. The Alarmin-ILC2-Eosinophil Amplification Circuit

The three alarmins converge on group 2 innate lymphoid cells (ILC2s), which respond to IL-33, TSLP, and IL-25 via cognate receptors, producing large quantities of IL-5 and IL-13 [7,46]. IL-5 prolongs eosinophil survival, enhances CCR3 expression, and augments CCL11 responsiveness, creating a positive-feedback amplification loop that can sustain eosinophilic inflammation in the absence of adaptive immune input [24]. IL-13 reinforces this loop by inducing eotaxin-3 production from esophageal epithelial cells and by suppressing barrier proteins such as DSG1, directly linking type 2 cytokine production to downstream eosinophil recruitment [39,47]. Furthermore, eosinophils themselves can produce IL-4 and IL-13 upon activation, positioning them as amplifiers rather than mere effectors of the alarmin cascade [9].

## 4. Eosinophil-to-Epithelial Cell Signaling: Mechanisms and Consequences

**Figure 2:**
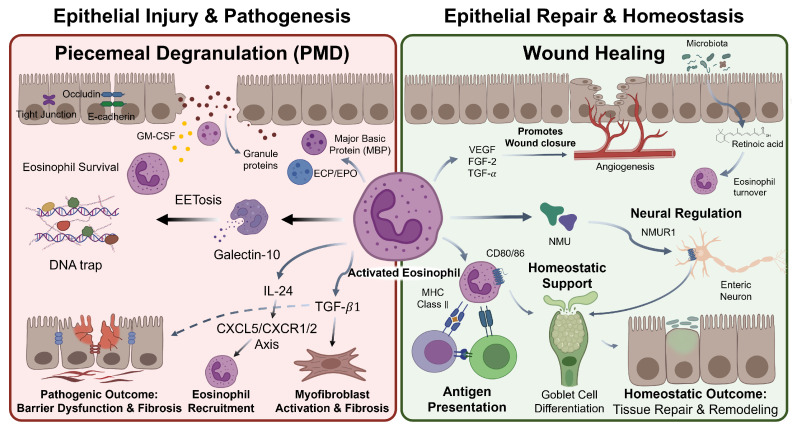
**Dual Roles of Activated Eosinophils at Mucosal Surfaces: Pathogenic Effector Functions and Homeostatic Support** (Left panel-Pathogenic) Activated eosinophils release granule proteins (MBP, ECP, EPO) and GM-CSF via piecemeal degranulation (PMD). EETosis releases galectin-10-decorated DNA traps. IL-24 disrupts tight junction integrity via the CXCL5/CXCR1/CXCR2 axis, and TGF-β1 activates myofibroblasts, collectively driving barrier dysfunction and subepithelial fibrosis. (Right panel-Homeostatic) Eosinophils support wound closure via VEGF, FGF-2, and TGF-α. Microbiota-derived retinoic acid regulates eosinophil turnover. Enteric neuronal NMU activates NMUR1-expressing eosinophils to promote goblet cell differentiation. Eosinophils expressing MHC class II, CD80, and CD86 serve as antigen-presenting cells. Abbreviations: PMD, piecemeal degranulation; MBP, major basic protein; ECP, eosinophil cationic protein; EPO, eosinophil peroxidase; GM-CSF, granulocyte-macrophage colony-stimulating factor; EET, eosinophil extracellular trap; IL, interleukin; TGF, transforming growth factor; CXCL5, C-X-C motif chemokine ligand 5; CXCR1/2, C-X-C chemokine receptor type 1/2; VEGF, vascular endothelial growth factor; FGF-2, fibroblast growth factor 2; NMU, neuromedin U; NMUR1, neuromedin U receptor 1; MHC II, major histocompatibility complex class II.

Eosinophils communicate with epithelial cells through a diverse repertoire of effector mechanisms, ranging from direct granule protein-mediated cytotoxicity to cytokine-driven transcriptional reprogramming and extracellular trap formation (Figure 2). This section focuses primarily on eosinophil-derived signals acting on epithelial cells; epithelium-to-eosinophil signaling is addressed in Section 3.

### 4.1. Granule Protein-Mediated Epithelial Injury

Eosinophil granule proteins are principal direct effectors of epithelial damage at mucosal surfaces. MBP-1, by virtue of its highly cationic nature (pI 10.9), disrupts the anionic lipid bilayer of epithelial cell membranes, increasing apical membrane conductance and impairing barrier integrity [4]. Motojima et al. demonstrated that MBP deposited on bronchial epithelial surfaces causes ciliostasis (disruption of ciliary beat frequency) at concentrations comparable to those found in bronchoalveolar lavage fluid (BALF) of asthmatic patients, implicating MBP in the mucus clearance defect of eosinophilic asthma [48]. ECP exerts cytotoxic effects through its pore-forming activity, rendering cell membranes permeable to other toxic molecules [4]. EPO generates hypobromous acid via halide-dependent oxidation, damaging epithelial glycocalyx proteins, and amplifying barrier disruption [4]. EDN, a secretory ribonuclease co-released alongside MBP and ECP during degranulation, contributes to the granule protein milieu at sites of mucosal injury but does not independently reduce transepithelial barrier function [49]. In a co-culture study of human eosinophils and T84 colonic epithelial cells, Furuta et al. demonstrated that cell-free eosinophil supernatants reduced transepithelial resistance (TER) and increased paracellular flux, with native MBP identified as the principal soluble mediator acting through downregulation of the tight junction protein occludin [49]. MBP-null mice were correspondingly protected from experimental oxazolone-induced colitis [49].

In EoE, extensive eosinophil degranulation occurs at sites of epithelial injury, depositing high local concentrations of granule proteins within the esophageal mucosa. Beyond direct cytolysis, Mulder et al. demonstrated that eosinophil-released MBP activates the calcium-sensing receptor (CaSR) on esophageal epithelial cells, triggering CaSR-dependent upregulation of fibroblast growth factor 9 (FGF9), an effect abrogated by siRNA-mediated CaSR knockdown [50]. FGF9 in turn drives epithelial proliferation via autocrine signaling through FGFR2 and FGFR3, and FGF9 concentrations in esophageal mucosal biopsies correlated positively with the degree of basal zone hyperplasia in EoE patients [50]. This MBP-CaSR-FGF9 axis defines a pathway through which granule proteins drive pathological epithelial remodeling independently of their direct cytotoxic activity.

### 4.2. Eosinophil-Derived Cytokines and Epithelial Modulation

Beyond granule proteins, eosinophils release a range of cytokines, including TGF-β1, IL-13, IL-24, and GM-CSF, that modulate epithelial behavior through both paracrine and autocrine mechanisms. Eosinophil-derived TGF-β1 is a major driver of subepithelial fibrosis and epithelial-mesenchymal transition (EMT) in eosinophilic inflammation [51]. In EoE, esophageal biopsies demonstrate elevated TGF-β1 and phosphorylated SMAD2/3 in eosinophil-rich lamina propria, with TGF-β1 expression correlating with subepithelial fibrosis and vascular activation [52]. TGF-β1 secreted by eosinophils activates subepithelial fibroblasts and promotes ECM deposition, contributing to esophageal stricture formation in severe EoE [52]. In the airway, eosinophil-derived TGF-β1 is activated by αvβ6 expressed on injured airway epithelial cells, generating a paracrine mechanism that directly links epithelial damage to fibrotic remodeling [53].

Wu et al. identified IL-24 as an eosinophil-derived mediator in allergic asthma that disrupts airway epithelial integrity [17]. Using OVA- and HDM-induced murine asthma models, they demonstrated that IL-24 is principally secreted by lung-infiltrating eosinophils and acts on airway epithelial cells to disrupt tight junction integrity via the CXCL5/CXCR1/CXCR2 axis [17]. IL-24 knockout mice exhibited reduced eosinophil infiltration, preserved epithelial barrier function, decreased fibrosis, and reduced mucus hypersecretion [17]. These findings establish an autocrine-paracrine circuit in which eosinophil-derived IL-24 promotes further eosinophil recruitment while simultaneously damaging the epithelial barrier. In the esophagus, co-culture of esophageal epithelial cells with eosinophils results in epithelial-derived GM-CSF release that sustains eosinophil activation and alters surface marker expression, establishing a contact-independent bidirectional circuit that operates independently of T cell input [15].

Eosinophil-derived IL-13 drives goblet cell metaplasia and MUC5AC hypersecretion in the airway through IL-4Rα/IL-13Rα1 signaling on airway epithelial cells [47,54]. Notably, IL-13-activated airway epithelial cell-derived mucus further triggers cytolytic eosinophil degranulation in a CD11b- and glycan-dependent manner, establishing a self-amplifying epithelial-mucin-eosinophil crosstalk loop in asthmatic airways [55]. In the esophagus, IL-13 produced by infiltrating eosinophils suppresses DSG1, amplifies periostin secretion from subepithelial fibroblasts, and promotes eotaxin-3 production, creating a cytokine amplification loop that sustains eosinophilic inflammation at the epithelial interface [39]. Beyond soluble mediators, eosinophils release extracellular vesicles (EVs), including exosomes, that mediate intercellular communication with structural lung cells independently of direct degranulation. Canas et al. demonstrated that exosomes derived from asthmatic eosinophils induce apoptosis in small airway epithelial cells, impair wound closure, and upregulate CCL26, TNF, and periostin expression in epithelial cells, while simultaneously promoting bronchial smooth muscle cell proliferation via ERK1/2 signaling, with none of these effects observed with exosomes from healthy eosinophils [56]. The potential of eosinophil-derived EVs as non-invasive biomarkers in EoE and concomitant atopic diseases has also been reviewed [57]. Collectively, the cytokine-mediated mechanisms described above converge with more direct forms of eosinophil effector function, including eosinophil extracellular trap formation, to shape the epithelial microenvironment.

### 4.3. Eosinophil Extracellular Traps (EETs) at the Epithelial Interface

Analogous to neutrophil extracellular traps (NETs), eosinophils can undergo a form of cell death termed ETosis, releasing chromatin decorated with eosinophil granule proteins to form eosinophil extracellular traps (EETs), as characterized in eosinophilic granulomatosis with polyangiitis (EGPA) [18]. Fukuchi et al. demonstrated in EGPA that EETosis requires reactive oxygen species (ROS) and peptidylarginine deiminase 4 (PAD4)-mediated histone citrullination, resulting in the cytolytic release of net-like EETs alongside free galectin-10 and membrane-bound intact granules [18]. Galectin-10, also known as the Charcot-Leyden crystal protein (CLCP), is the most abundant protein in human eosinophil granules and has long served as a histological marker of eosinophilic tissue infiltration. Beyond its role as a structural component of EETs, extracellularly deposited galectin-10 has been shown to form Charcot-Leyden crystals at mucosal surfaces, where crystal deposition acts as a danger signal that amplifies innate immune responses and perpetuates eosinophilic inflammation. In CRSwNP, galectin-10 expression in nasal polyp tissue correlates directly with disease severity as assessed by Clinical-Cytological Grading, with co-localization in infiltrating eosinophils and mast cells, establishing galectin-10 as both a mechanistic contributor to and a biomarker of mucosal eosinophilic pathology [58]. Ueki et al. demonstrated that EETosis mediates the lytic release of free, secretion-competent eosinophil granules that retain the capacity to degranulate upon stimulation with eotaxin-1, establishing that EETs carry functionally active granule proteins capable of further mediator release at sites of deposition [30].

At the mucosal surface, EETs damage epithelial cells through deposition of cationic granule proteins at high local concentrations [18,30]. In severe asthma, Lu et al. demonstrated that EETs drive asthma progression by activating pulmonary neuroendocrine cells via the CCDC25–ILK–PKCα-CRTC1 pathway, which amplifies allergic immune responses through neuropeptide and neurotransmitter release [59]. Choi et al. established that EETs activate ILC2s by stimulating airway epithelial cytokine release (IL-33, TSLP) in severe asthma, providing a mechanistic basis by which eosinophil cell death amplifies the alarmin-ILC2-eosinophil circuit [60]. In CRSwNP, Gevaert et al. demonstrated that *S. aureus* colonization at sites of epithelial barrier defects directly induces EET formation in nasal polyp tissue, implicating eosinophil ETosis in the pathological response to bacterial colonization in upper airway disease [61].

### 4.4. Eosinophil-Mediated Epithelial Repair and Homeostasis

Not all eosinophil–epithelial interactions are destructive. Eosinophils also produce mediators with tissue-reparative properties including VEGF, FGF-2, and TGF-α that support angiogenesis, fibroblast proliferation, and wound closure [6,9]. In the SI, NMUR1-expressing eosinophils activated by enteric neuronal NMU directly promote goblet cell differentiation in organoid co-culture systems and maintain intestinal barrier immunity against helminth infection [16]. Xenakis et al. further demonstrated that intestinal eosinophils constitutively express antigen presentation markers (MHC class II, CD80, CD86), defining two phenotypically distinct subsets and positioning them as bidirectional mediators capable of acquiring lumen-derived antigens and presenting them to adaptive immune system [62]. Cao et al. demonstrated that Faecalibaculum rodentium remodels retinoic acid signaling in intestinal eosinophils, maintaining an eosinophil population that suppresses intraepithelial lymphocyte-derived IFN-γ to govern duodenal epithelial homeostasis [63].

Beyond cytokine and lipid mediator-based communication, nerves constitute a third cellular axis in the eosinophil–epithelial regulatory network, forming bidirectional loops that operate across airway, gastrointestinal, and cutaneous mucosal compartments. In the small intestinal mucosa, the enteric neuron-eosinophil NMU-NMUR1 axis described above [16] exemplifies how neuropeptide signals can directly program eosinophil effector function in a tissue-specific manner, a principle that extends across mucosal compartment. In the airway, eosinophils and mast cells are found in close physical contact with sensory nerve bundles in fatal asthma tissue, where sensory neuropeptides including calcitonin gene-related peptide (CGRP) are elevated, suggesting that neuroimmune interactions contribute to the severity of eosinophilic airway disease [64]. EET formation by airway eosinophils further activates pulmonary neuroendocrine cells through the CCDC25-ILK-PKCα-CRTC1 pathway, amplifying allergic immune responses via neuropeptide release [59]. At the systemic level, sympathetic neurons modulate eosinophil activation through the CCL11–CCR3 axis and beta2 adrenergic receptor signaling, establishing a stress-responsive neuroimmune circuit that exacerbates type 2 mucosal inflammation [34]. Collectively, these epithelial–nerve–eosinophil interactions define a tripartite regulatory axis that amplifies or resolves mucosal inflammation in a tissue- and context-dependent manner.

## 5. Eosinophil–Epithelial Crosstalk in Airway Mucosal Disease

The mechanisms described in Section 4 manifest with distinct tissue-specific consequences across eosinophilic airway diseases. This section examines how eosinophil–epithelial crosstalk drives pathology in eosinophilic asthma and CRSwNP.

### 5.1. Eosinophilic Asthma

Asthma is a heterogeneous syndrome encompassing distinct endotypes defined by their underlying immunological mechanisms and cellular contributors [65]. The type 2-high eosinophilic endotype, driven by IL-4, IL-5, and IL-13 and characterized by ILC2 activation, mast cell involvement, and prominent eosinophil recruitment, represents the predominant context in which eosinophil–epithelial crosstalk operates as a pathogenic mechanism [7,46]. In contrast, type 2-low endotypes including neutrophilic and paucigranulocytic asthma, are driven primarily by IL-17 and IFN-γ, with a correspondingly limited role for eosinophil–epithelial interactions [65]. In eosinophilic asthma, driven by a type 2-high immune milieu rich in eosinophils, mast cells, and ILC2s [7,46], the localization of eosinophils within the airway epithelium is mechanistically linked to AHR and declining lung function. Al-Shaikhly et al. used stereological analysis of bronchoscopic biopsies to demonstrate that intraepithelial eosinophil localization is specifically associated with endogenous AHR and IL-5 expression, whereas subepithelial eosinophils were associated with type 2 inflammation more broadly, establishing a location-dependent functional distinction with implications for disease phenotyping [66]. Intraepithelial eosinophils release MBP-1 that antagonizes inhibitory muscarinic M2 receptors on parasympathetic neurons, potentiating bronchoconstriction and AHR [67]. Al-Shaikhly et al. further demonstrated that intraepithelial eosinophils interact with intraepithelial mast cells through cysteinyl leukotriene and IL-33-mediated ST2 upregulation to amplify airway inflammation [66]. Concurrently, eosinophil-derived IL-13 drives goblet cell metaplasia and MUC5AC hypersecretion through IL-4Rα signaling on airway epithelial cells, directly contributing to mucus hypersecretion as a cardinal feature of eosinophilic asthma [47,54,68].

Raggi et al. described a DUOX1-mediated eosinophil–airway epithelial cell crosstalk mechanism in a murine allergic asthma model [69]. Eosinophils activated by IL-33 upregulate DUOX1 expression in airway epithelial cells; DUOX1-generated H_2_O_2_ then drives non-canonical IL-33 secretion, creating a feedback loop that sustains eosinophil–epithelial crosstalk in a contact-independent manner [69]. DUOX1 has been detected in airway epithelial cells from patients with allergic asthma and CRSwNP, suggesting clinical relevance of this eosinophil-driven oxidative loop [69].

### 5.2. Airway Remodeling

Beyond acute bronchospasm and epithelial activation, chronic eosinophil–epithelial interactions drive irreversible structural changes. Airway remodeling, characterized by subepithelial fibrosis, goblet cell metaplasia, smooth muscle hypertrophy, and angiogenesis, is a hallmark of chronic eosinophilic asthma. Intraepithelial eosinophil accumulation has been associated with an altered mucus-repair phenotype, TGF-β2 overexpression, and basement membrane reticular layer thickening [10]. Eosinophil-derived TGF-β1 is activated by αvβ6 on injured airway epithelial cells, generating a paracrine loop that directly links epithelial injury to subepithelial fibrosis [53]. Anti-IL-5 antibody treatment in asthmatic patients reduces eosinophil-derived TGF-β1, epithelial αvβ6 expression, and basement membrane tenascin-C, providing clinical evidence that eosinophil depletion directly attenuates the downstream structural effects of eosinophil–epithelial crosstalk [10].

The clinical impact of anti-eosinophil therapy on airway remodeling was further confirmed by Domvri et al., who demonstrated in the MESILICO study that 12 months of mepolizumab treatment significantly reduced sub-basement membrane thickness, airway smooth muscle area, and intraepithelial eosinophil counts in patients with severe late-onset eosinophilic asthma, with the extent of airway smooth muscle reduction directly correlating with submucosal eosinophil depletion [70].

### 5.3. Chronic Rhinosinusitis with Nasal Polyps (CRSwNP)

Eosinophil–epithelial crosstalk in the upper airways shares mechanistic features with asthma but operates within a distinct anatomical and immunological context. CRSwNP is an eosinophil-dominated type 2 inflammatory disease of the sinonasal mucosa characterized by pronounced barrier dysfunction. Nasal polyp epithelium exhibits reduced tight junction protein expression alongside elevated TSLP production and, in a subset of patients, IL-33, though the latter remains a subject of ongoing debate [71]. IL-13 produced within the type 2 inflammatory milieu of nasal polyps directly induces eotaxin-3 expression by sinonasal epithelial cells, and tissue eotaxin-3 levels correlate positively with eosinophil cationic protein concentrations and disease severity, establishing a cytokine-driven epithelial amplification loop for eosinophil recruitment [72]. Hisamatsu et al. demonstrated that MBP causes ciliostasis and disrupts the surface profile of human nasal sinus mucosa in vitro at concentrations relevant to eosinophilic inflammation, with allergic mucosal specimens showing heightened sensitivity, implicating MBP-mediated epithelial damage in the mucociliary clearance defect of CRSwNP [73]. In CRSwNP, *Staphylococcus aureus* colonization at sites of epithelial barrier defects directly induces EET formation in nasal polyp tissue alongside elevated periostin and IL-5 levels, implicating eosinophil ETosis in the amplification of the type 2 mucosal inflammatory response [61]. A recent multi-scale single-cell and spatial transcriptomics study of human CRSwNP tissue further mapped the immune-epithelial landscape of nasal polyp formation at single-cell resolution, revealing macrophage-eosinophil recruitment programs, mast cell enrichment, and a distinct basal cell trajectory linked to polyp formation, with immune–epithelial interactions validated across more than 100 individuals [74].

## 6. Eosinophil–Epithelial Crosstalk in Gastrointestinal Disease

Eosinophil–epithelial interactions in the gastrointestinal tract span a spectrum from homeostatic tissue maintenance to frank inflammatory pathology. This section examines these interactions in the context of intestinal homeostasis, EoE, and IBD.

### 6.1. Homeostatic Intestinal Eosinophil–Epithelial Interactions

Under homeostatic conditions, eosinophils are the dominant granulocytes of the SI lamina propria, constituting up to 20% of lamina propria CD45+ cells in the jejunum [11]. This homeostatic eosinophil presence is actively maintained by intestinal epithelial cells, which serve as the principal source of constitutive eotaxin-1 production; selective loss of IKKβ signaling in intestinal epithelial cells reduces lamina propria eotaxin levels and eosinophil numbers, and impairs the effector phase of oral food allergy, establishing IEC-derived eotaxin as a critical regulator of intestinal eosinophil homeostasis [22]. In addition to these villous homeostasis functions, intestinal eosinophils promote IgA production and maintenance of IgA-secreting plasma cells through mechanisms involving direct cellular interactions and eosinophil-derived mediators [75]. Xenakis et al. demonstrated that SI resident eosinophils constitutively express antigen presentation markers MHC class II, CD80, and CD86, defining two phenotypically distinct subsets and positioning intestinal eosinophils as antigen-presenting cells capable of relaying luminal signals to the adaptive immune cells [62]. Cao et al. used germ-free and gnotobiotic mouse models to demonstrate that F. rodentium remodels retinoic acid signaling in intestinal eosinophils, maintaining an eosinophil population that suppresses intraepithelial lymphocyte-derived IFN-γ to govern duodenal epithelial homeostasis [63]. These findings suggest that gut dysbiosis may deregulate eosinophil homeostatic functions in ways that contribute to epithelial barrier breakdown independently of overt inflammation.

### 6.2. Eosinophilic Esophagitis: The Bidirectional Crosstalk Paradigm

While eosinophils serve homeostatic roles in the normal intestine, their accumulation in tissues that are not normally eosinophilic such as the esophagus, defines the pathological crosstalk characteristic of eosinophilic gastrointestinal disorders. EoE is the prototypic eosinophil–epithelial crosstalk disease, defined by the accumulation of ≥15 eosinophils per high-power field in esophageal mucosa, a tissue normally devoid of eosinophils [76]. The molecular basis for esophageal eosinophilia was established through genetic studies: a single-nucleotide polymorphism in the 3’ untranslated region of CCL26 (eotaxin-3) increases esophageal eosinophil recruitment by augmenting CCL26 transcription [77]. Additionally, loss-of-function mutations in FLG impair epithelial barrier formation, increasing allergen sensitization and disease susceptibility [38]. Sherrill et al. demonstrated that DSG1, a desmosomal adhesion protein whose expression is suppressed by IL-13, regulates esophageal epithelial barrier function and immune responses, providing a mechanistic explanation for the IL-13-driven barrier-failure–eosinophilia feedback loop characteristic of EoE [39].

The bidirectional nature of EoE eosinophil–epithelial crosstalk was established by Dunn et al. in a non-contact co-culture system demonstrating that eosinophils prolong their own survival in response to epithelial-derived GM-CSF while simultaneously altering the epithelial cell transcriptome, upregulating genes associated with cell proliferation, ECM remodeling, and wound healing [15]. This circuit operates independently of T cells. The large-scale single-cell atlas of EoE by Ding et al. (421,312 cells from 7 healthy and 15 EoE participants) mapped the cellular ecosystem of the diseased esophagus, revealing active disease-specific enrichment of ALOX15+ macrophages, cycling mast cells, and PRDM16+ dendritic cells expressing the EoE risk gene ATP10A [14]. Ligand–receptor interaction analysis identified epithelial-derived CCL11, CCL24, and CCL26 as the primary eosinophil recruitment signals, and resolution of inflammation during remission was characterized by downregulation of these chemoattractants in a cell-type-specific manner [14].

### 6.3. Inflammatory Bowel Disease

In contrast to EoE, where eosinophil accumulation occurs in a normally eosinophil-free tissue, IBD represents a context in which baseline intestinal eosinophilia becomes pathologically amplified. In IBD, tissue eosinophilia is a consistent feature of active mucosal pathology. In UC, lamina propria cells produce elevated IL-5 and IL-13, cytokines that promote eosinophil survival and eotaxin-mediated recruitment [78,79]. The direct impact of eosinophils on intestinal epithelial barrier function was demonstrated by Furuta et al., who showed that eosinophil-T84 cell co-cultures produced significant TER reduction and increased paracellular flux via MBP and EDN, directly implicating eosinophil granule proteins in the barrier disruption of IBD [49]. Lampinen et al. demonstrated that eosinophils from UC patients in apparent clinical remission retain an activated degranulation phenotype compared to healthy controls, suggesting that persistent subclinical eosinophil activation at the epithelial interface may contribute to maintenance of mucosal barrier dysfunction during remission [13]. The eotaxin–IL-13–periostin axis has been proposed as a biomarker of active eosinophilic mucosal inflammation in UC, with elevated serum periostin correlating with disease activity [80,81]. In contrast to UC, where eosinophilia is a defining feature, mucosal eosinophil infiltration in CD is more heterogeneous and less pronounced, reflecting the predominantly Th1/Th17 immunopathology of the disease. Nevertheless, eosinophil degranulation has been documented at sites of crypt injury in active CD, suggesting a context-dependent contribution to epithelial barrier disruption.

## 7. Shared Mechanisms and Tissue-Specific Differences

**Figure 3:**
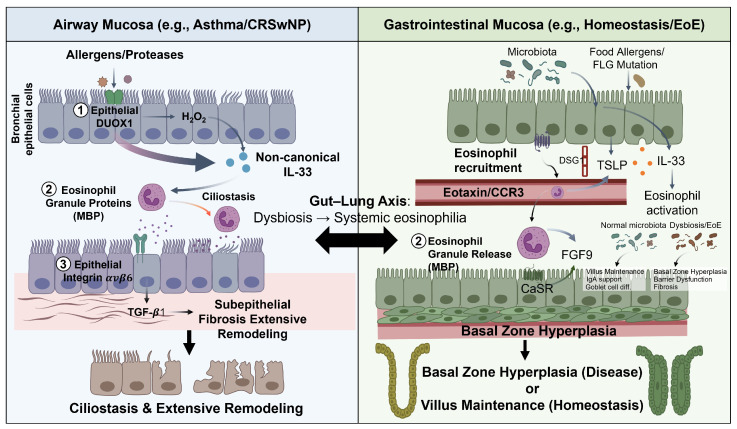
**Tissue-Specific Eosinophil–Epithelial Crosstalk in Airway and Gastrointestinal Mucosal Disease** (Left panel-Airway mucosa) In asthma and CRSwNP, allergen/protease-activated bronchial epithelial DUOX1 generates H_2_O_2_ that drives non-canonical IL-33 secretion (1) MBP induces ciliostasis and barrier dysfunction (2) Epithelial integrin αvβ6 activates eosinophil-derived TGF-β1, driving subepithelial fibrosis (3) These mechanisms collectively result in ciliostasis and extensive airway remodeling. (Right panel-Gastrointestinal mucosa) Under homeostasis, microbiota-dependent IL-33 and TSLP support eosinophil activation via the eotaxin/CCR3 axis. In EoE, food allergens and FLG mutations drive barrier dysfunction and DSG1 suppression, amplifying eosinophil recruitment. MBP activates the CaSR–FGF9 axis, driving basal zone hyperplasia. Homeostatic eosinophils maintain villus architecture, IgA production, and goblet cell differentiation, whereas dysbiosis and EoE shift the balance toward barrier dysfunction and fibrosis. Abbreviations: CRSwNP, chronic rhinosinusitis with nasal polyps; DUOX1, dual oxidase 1; H_2_O_2_, hydrogen peroxide; MBP, major basic protein; TGF-β1, transforming growth factor beta 1; αvβ6, integrin alpha-v beta-6; IL, interleukin; TSLP, thymic stromal lymphopoietin; CCR3, C-C chemokine receptor type 3; FLG, filaggrin; DSG1, desmoglein-1; CaSR, calcium-sensing receptor; FGF9, fibroblast growth factor 9; EoE, eosinophilic esophagitis; IgA, immunoglobulin A.

A comparison of eosinophil–epithelial crosstalk across airway and GI mucosal compartments reveals shared molecular principles alongside important tissue-specific differences (Figure 3). The alarmin triad (IL-33, TSLP, IL-25) is a common initiating signal in both compartments, but with tissue-specific weighting: IL-33 is the dominant microbiota-responsive alarmin in the SI [11], whereas TSLP plays prominent roles in esophageal EoE [44] and airway disease [43]. The downstream ILC2-eosinophil amplification loop through IL-5 and IL-13, the IL-5/IL-5Rα axis for eosinophil survival, and the eotaxin/CCR3 axis for tissue recruitment are conserved across compartments [1,24].

Mechanistic differences emerge at the level of downstream epithelial pathways. In the airway, MBP-mediated ciliostasis [48] and DUOX1-driven IL-33 secretion [69] are particularly prominent. MBP-mediated airway hyperresponsiveness is a further airway-specific mechanism: beyond direct epithelial injury, MBP antagonizes inhibitory muscarinic M2 receptors on parasympathetic neurons, potentiating bronchoconstriction through dysfunctional neuronal signaling, and directly affects airway smooth muscle contractility [67], representing a neuroepithelial axis with no equivalent in the GI tract. In the esophagus, the MBP-CaSR-FGF9 basal zone hyperplasia axis [50] and TGF-β1-driven EMT dominate the remodeling phenotype [51,52]. The SI exhibits a unique homeostatic circuit in which eosinophils support villous architecture and goblet cell differentiation through NMUR1-NMU neuroimmune signaling [16] and microbiota-dependent IL-33 mechanisms [11], functional programs that have no direct equivalent in the airway. EET-mediated epithelial damage is documented in both lower airway (asthma) and upper airway (CRSwNP) disease, suggesting ETosis as a shared response to severe barrier disruption [59,60,61].

The role of the microbiome in modulating eosinophil–epithelial crosstalk is more pronounced in the GI tract, where commensal bacteria directly condition eosinophil phenotype through retinoic acid signals [63]. Extending this paradigm across compartments, Burrows et al. demonstrated that a gut commensal protozoan remotely shapes pulmonary eosinophilia through a tripartite ILC2-T cell-B cell network, exacerbating allergic airway inflammation while simultaneously conferring protection against pulmonary Mycobacterium tuberculosis dissemination [82]. This dual outcome illustrates the context-dependent consequences of microbiome-driven eosinophil programming across mucosal compartments. Gut dysbiosis may therefore deregulate eosinophil homeostatic functions in ways that amplify epithelial barrier breakdown, offering a mechanistic framework for the epidemiological associations between altered gut microbiota and mucosal inflammatory diseases [11,83].

## 8. Therapeutic Implications

### 8.1. Targeting the Alarmin–Eosinophil Axis

The eosinophil–epithelial crosstalk axis provides multiple actionable therapeutic targets. Tezepelumab, a monoclonal antibody targeting TSLP, reduces blood and sputum eosinophilia across all asthma phenotypes in the NAVIGATOR phase 3 trial, including patients with low blood eosinophil counts, demonstrating that upstream alarmin blockade interrupts the eosinophil–epithelial circuit at its initiation [84]. A companion mechanistic study (CASCADE) showed that tezepelumab significantly reduced airway mucosal eosinophilia, subepithelial thickness, and airway hyperresponsiveness compared to placebo, providing direct tissue-level evidence that TSLP drives the structural consequences of eosinophil–epithelial crosstalk [85]. Anti-IL-33 antibodies itepekimab showed significant reduction in asthma control loss events alongside eosinophil lowering in a phase 2 trial [86], supporting IL-33 as a valid alarmin target in type 2 airway inflammation.

### 8.2. Direct Eosinophil Depletion

Anti-IL-5 therapies directly deplete tissue eosinophils and interrupt the downstream consequences of eosinophil–epithelial crosstalk. Consistent with this mechanism, IL-5 has been shown to act primarily through transit amplification of bone marrow eosinophil progenitors rather than terminal maturation, providing a mechanistic basis for why IL-5 blockade selectively reduces eosinophil numbers without disrupting the broader myeloid differentiation program [87]. Ortega et al. (MENSA trial) demonstrated that subcutaneous mepolizumab reduced asthma exacerbation rates by 53% versus placebo and reduced blood and sputum eosinophils in patients with severe eosinophilic asthma [88]. Pavord et al. (DREAM trial) confirmed that exacerbation reduction correlated with baseline blood eosinophil count and prior exacerbation history, establishing blood eosinophilia as a predictive biomarker of mepolizumab response [89]. Consistent with the mechanistic role of eosinophils in airway remodeling, anti-IL-5 treatment reduces eosinophil-derived TGF-β1, αvβ6 expression, and basement membrane tenascin-C in bronchial biopsies [10], and the MESILICO study confirmed significant reduction in sub-basement membrane thickness and airway smooth muscle area after 12 months of mepolizumab in severe eosinophilic asthma [70]. Benralizumab (anti-IL-5Rα), which depletes eosinophils via ADCC, achieved significant exacerbation reduction in the SIROCCO and CALIMA phase 3 trials [90,91].

### 8.3. Dual Pathway Blockade with Dupilumab

Dupilumab targets the shared IL-4Rα receptor subunit and suppresses multiple arms of eosinophil–epithelial crosstalk simultaneously. By blocking IL-4 and IL-13 signaling, dupilumab prevents DSG1 suppression and CCL26 upregulation in EoE, reduces eotaxin-3-driven eosinophil recruitment, and restores epithelial barrier gene expression [39,77]. A phase 3 randomized trial demonstrated that dupilumab significantly reduced esophageal eosinophil counts, histologic scores, and dysphagia symptoms in EoE, leading to FDA approval and establishing dupilumab as the first biologic approved for this indication [92,93]. Dupilumab is also approved for severe asthma and CRSwNP, representing the broadest-spectrum anti-eosinophilic biologic across mucosal compartments [92,94,95].

### 8.4. Emerging Targets

Novel mediators identified in eosinophil–epithelial crosstalk are generating new therapeutic candidates. The IL-24 pathway uncovered by Wu et al. [17] represents a potential target whose blockade could simultaneously reduce eosinophil recruitment and preserve epithelial barrier integrity in eosinophilic asthma. Anti-Siglec-8 (lirentelimab), which depletes eosinophils and inhibits mast cells via an inhibitory Siglec-8 signaling mechanism, has demonstrated efficacy in reducing eosinophil counts in eosinophilic gastritis and duodenitis in phase 2 trials [96]. Bispecific antibodies targeting TSLP/IL-33 or TSLP/IL-4/IL-13 are under preclinical and early clinical development, aiming to provide synergistic suppression of multiple nodes in the alarmin–eosinophil amplification loop [97]. DUOX1 inhibition is an emerging airway-specific target given its role in sustaining the eosinophil–DUOX1–IL-33 feedback circuit identified by Raggi et al. [69].

## 9. Future Perspectives and Conclusions

Several key questions remain unresolved. First, the functional consequences of eosinophil heterogeneity at human mucosal surfaces require systematic investigation. The transcriptomic profiling approach applied in murine models [16] and the single-cell atlas of the EoE mucosa [14] needs extension to human airway and intestinal tissue under both homeostatic and inflammatory conditions. Whether NMUR1+ eosinophils identified in the murine SI have human homologs with similar goblet cell-promoting functions [16] is a critical unanswered question. Second, the dual role of EETs, simultaneously protective against pathogens and damaging to epithelial surfaces, demands resolution using spatially and temporally resolved imaging methods that can distinguish beneficial from pathological ETosis [18,59,60].

Spatially resolved transcriptomics platforms, which preserve the spatial context of cell–cell interactions, have the potential to substantially advance understanding of eosinophil–epithelial proximity at mucosal surfaces. Combined with multiplexed protein imaging, spatial transcriptomics could definitively map the distance-dependent signaling gradients between degranulating eosinophils and adjacent epithelial cells in health and disease. Primary epithelial organoid systems co-cultured with defined eosinophil populations provide an accessible human experimental model that could connect murine genetic findings to clinical observations, as demonstrated by the NMUR1-organoid co-culture experiments of Li et al. [16]. The microbiome-eosinophil–epithelial axis also represents an underexplored therapeutic avenue: targeting microbiota compositions that optimize eosinophil homeostatic programming [63] could represent a preventive strategy for EoE and IBD.

In conclusion, eosinophil–epithelial crosstalk at mucosal barriers constitutes a dynamic, bidirectional regulatory axis that is essential for tissue homeostasis and a major pathogenic driver when dysregulated. The alarmin triad (IL-33, TSLP, IL-25), the ILC2-eosinophil amplification circuit, and the diverse portfolio of eosinophil-derived mediators including granule proteins, TGF-β1, IL-13, IL-24, and EETs, collectively mediate this crosstalk. The airway and GI compartments share core mechanisms but differ importantly in the homeostatic roles and pathological signatures of eosinophil–epithelial interactions. A comprehensive mechanistic understanding of this axis across mucosal compartments is essential for developing targeted therapies that can precisely interrupt the eosinophil–epithelial circuit without compromising the homeostatic functions integral to mucosal immunity.

## Data Availability

No new data were created or analyzed in this study.
